# Metabolic and lifestyle risk factors for acute pancreatitis in Chinese adults: A prospective cohort study of 0.5 million people

**DOI:** 10.1371/journal.pmed.1002618

**Published:** 2018-08-01

**Authors:** Yuanjie Pang, Christiana Kartsonaki, Iain Turnbull, Yu Guo, Ling Yang, Zheng Bian, Yiping Chen, Iona Y. Millwood, Fiona Bragg, Weiwei Gong, Qinai Xu, Quan Kang, Junshi Chen, Liming Li, Michael V. Holmes, Zhengming Chen

**Affiliations:** 1 Clinical Trial Service Unit & Epidemiological Studies Unit (CTSU), Nuffield Department of Population Health, University of Oxford, Oxford, United Kingdom; 2 Medical Research Council Population Health Research Unit (MRC PHRU), Nuffield Department of Population Health, University of Oxford, Oxford, United Kingdom; 3 Chinese Academy of Medical Sciences, Beijing, China; 4 Provincial Center for Disease Control, Zhejiang Province, China; 5 Nangang Center for Disease Control, Heilongjiang Province, China; 6 National Center for Food Safety Risk Assessment, Beijing, China; 7 School of Public Health, Peking University, Beijing, China; 8 National Institute for Health Research Oxford Biomedical Research Centre, Oxford University Hospital, Oxford, United Kingdom; Chinese University of Hong Kong, CHINA

## Abstract

**Background:**

Little prospective evidence exists about risk factors and prognosis of acute pancreatitis in China. We examined the associations of certain metabolic and lifestyle factors with risk of acute pancreatitis in Chinese adults.

**Methods and findings:**

The prospective China Kadoorie Biobank (CKB) recruited 512,891 adults aged 30 to 79 years from 5 urban and 5 rural areas between 25 June 2004 and 15 July 2008. During 9.2 years of follow-up (to 1 January 2015), 1,079 cases of acute pancreatitis were recorded. Cox regression was used to estimate adjusted hazard ratios (HRs) for acute pancreatitis associated with various metabolic and lifestyle factors among all or male (for smoking and alcohol drinking) participants. Overall, the mean waist circumference (WC) was 82.1 cm (SD 9.8) cm in men and 79.0 cm (SD 9.5) cm in women, 6% had diabetes, and 6% had gallbladder disease at baseline. WC was positively associated with risk of acute pancreatitis, with an adjusted HR of 1.35 (95% CI 1.27–1.43; *p* < 0.001) per 1-SD-higher WC. Individuals with diabetes or gallbladder disease had HRs of 1.34 (1.07–1.69; *p* = 0.01) and 2.42 (2.03–2.88; *p* < 0.001), respectively. Physical activity was inversely associated with risk of acute pancreatitis, with each 4 metabolic equivalent of task (MET) hours per day (MET-h/day) higher physical activity associated with an adjusted HR of 0.95 (0.91–0.99; *p* = 0.03). Compared with those without any metabolic risk factors (i.e., obesity, diabetes, gallbladder disease, and physical inactivity), the HRs of acute pancreatitis for those with 1, 2, or ≥3 risk factors were 1.61 (1.47–1.76), 2.36 (2.01–2.78), and 3.41 (2.46–4.72), respectively (*p* < 0.001). Among men, heavy alcohol drinkers (≥420 g/week) had an HR of 1.52 (1.11–2.09; *p* = 0.04, compared with abstainers), and current regular smokers had an HR of 1.45 (1.28–1.64; *p* = 0.02, compared with never smokers). Following a diagnosis of acute pancreatitis, there were higher risks of pancreatic cancer (HR = 8.26 [3.42–19.98]; *p* < 0.001; 13 pancreatic cancer cases) and death (1.53 [1.17–2.01]; *p* = 0.002; 89 deaths). Other diseases of the pancreas had similar risk factor profiles and prognosis to acute pancreatitis. The main study limitations are ascertainment of pancreatitis using hospital records and residual confounding.

**Conclusions:**

In this relatively lean Chinese population, several modifiable metabolic and lifestyle factors were associated with higher risks of acute pancreatitis, and individuals with acute pancreatitis had higher risks of pancreatic cancer and death.

## Abstract 2

In a prospective cohort study including 0.5 million Chinese adults, Yuanjie Pang and colleagues investigate whether factors such as adiposity, diabetes, drinking, smoking, and physical activity are associated with risk of acute pancreatitis.

## Author summary

**Why was this study done?:**

- Smoking, alcohol, and gallbladder disease are risk factors for acute pancreatitis in Western populations.
- The relevance of certain lifestyle and metabolic factors (e.g., adiposity, diabetes, and physical activity) for acute pancreatitis has not been properly investigated in East Asian populations.
- In China, there is limited prospective evidence on risk factors for acute pancreatitis.

**What did the researchers do and find?:**

- A total of 512,891 Chinese adults were recruited from 5 urban and 5 rural areas across China in 2004–2008; participants completed a detailed questionnaire interview and underwent physical measurements and blood tests, and we then tracked their health until 1 January 2015 (median 9.2 years).
- Individuals with diabetes, gallbladder disease, or higher levels of adiposity were at a significantly higher risk of developing acute pancreatitis, while those with higher levels of physical activity had a lower risk of acute pancreatitis. Among men, smoking and heavy alcohol drinking were important risk factors for acute pancreatitis.
- Participants with acute pancreatitis were at higher risk of death and developing pancreatic cancer.

**What do these findings mean?:**

- To our knowledge, this is the first prospective study investigating lifestyle and metabolic risk factors for acute pancreatitis in the Chinese population.
- The modifiable nature of the risk factors suggests that, assuming cause and effect, many cases of acute pancreatitis in Chinese individuals might be prevented with modifications to lifestyle.
- The strong link between acute pancreatitis and pancreatic cancer, as well as their shared risk factors (i.e., smoking, heavy drinking, adiposity, diabetes), suggests that acute pancreatitis and pancreatic cancer may share similar aetiologies.

## Introduction

Acute pancreatitis is one of the most common gastrointestinal causes of hospital admission, with a case fatality rate ranging from 2% to 10%.[1] Chronic pancreatitis, although lower in prevalence than acute pancreatitis, has profound effects on quality of life [1]. Patients with acute or chronic pancreatitis are at higher risk of death and of developing pancreatic cancer [2,3]. Globally, the reported incidence of acute pancreatitis ranges from 4 to 45 per 100,000 persons, and in most high-income countries, it is increasing by about 2% to 4% annually [1]. In China, there are no large population-based data about the burden of pancreatitis available, although a recent Global Burden of Disease report suggested that the age-standardised death rate from pancreatitis in China has remained unchanged since 1990 [4]. Several important risk factors for pancreatitis—including gallstone disease, smoking, and heavy alcohol drinking—are known [5,6], although approximately 20% of pancreatitis cases are idiopathic (i.e., the aetiology remains undetermined) [5,6]. Other possible risk factors include obesity, diabetes, hypertriglyceridemia, and use of certain medications (e.g., oestrogens, steroids) [1,5,6]. Some of these risk factors for acute pancreatitis are also risk factors for pancreatic cancer, including smoking, heavy alcohol drinking, adiposity, and diabetes [7]. Although various mechanisms have been hypothesised, the aetiology of acute pancreatitis is still poorly understood [1].

Previous prospective cohort studies have shown that smoking and alcohol are associated with risk of pancreatitis, regardless of the subtype [1,5,6]. Similarly, adiposity and diabetes were associated with higher risk of acute pancreatitis [1,5,6]. However, previous evidence involved mainly Western populations, in which—in contrast to those in China and other East Asian countries—a greater proportion of people are overweight or affected by obesity [8]. Appropriate understanding of the modifiable metabolic and lifestyle risk factors for acute pancreatitis in East Asian populations, which may differ from those in Western populations due to the different associations between adiposity and insulin resistance [9], can inform disease prevention and treatment. We examined relevant data in the prospective China Kadoorie Biobank (CKB) of 0.5 million adults to assess (1) whether adiposity and diabetes were positively associated with risk of acute pancreatitis, (2) whether smoking and alcohol were associated with higher risk of acute pancreatitis among men, and (3) whether individuals with acute pancreatitis had higher risks of death and developing pancreatic cancer. Moreover, we also examined the joint effects of metabolic risk factors on risk of acute pancreatitis.

## Methods

### Study population

The CKB design, survey methods, and population characteristics have been previously described [10]. In summary, 512,891 participants (210,222 men and 302,669 women) aged between 30 and 79 years were recruited into CKB from 5 urban and 5 rural locations in China from 25 June 2004 to 15 July 2008. The 10 study areas provide diversity in patterns of risk exposures and diseases but also take into account population stability, the quality of morbidity and mortality registries, and other factors [10]. A total of 1,801,167 persons were originally selected for recruitment, and the overall population response rate was 28%. The CKB study was approved by the Ethical Review Committee of the Chinese Centre for Disease Control and Prevention and the Oxford Tropical Research Ethics Committee, University of Oxford. All participants eligible for this study had completed a written informed consent form. All methods were performed in accordance with relevant guidelines and regulations.

At local study assessment clinics, participants completed a laptop-based questionnaire administered by a trained health worker. The questionnaire covered information on sociodemographic characteristics, lifestyle (smoking, alcohol intake, diet, physical activity), personal and family medical history, and current medication. Participants also went through a physical examination by trained technicians using calibrated instruments with standard protocols. Physical measurements included height, weight, hip circumference (HC), waist circumference (WC), bio-impedance, lung function, blood pressure, and heart rate [10]. In addition, participants provided a 10-ml nonfasting blood sample, with the time since participants last ate recorded.

### Data collection on metabolic and lifestyle factors

General adiposity measures were body mass index (BMI), body fat percentage (%BF), and height-adjusted weight; central adiposity measures were WC, HC, and waist-to-hip ratio (WHR). Standing height was measured using a stadiometer. Weight and %BF were measured using a body composition analyser (TANITA-TBF-300GS; Tanita Corporation, Tokyo, Japan). Weight of clothing was subtracted from body weight according to the season (ranging from 0.5 kg in summer to 2.0–2.5 kg in winter). %BF was the fraction of total weight that was estimated to be fat weight using proprietary algorithms. WC and HC were measured using a nonstretchable tape. WHR was the ratio of WC to HC. BMI at baseline was defined as the measured weight (kg) divided by the square of the measured height (m). BMI at age 25 (BMI25) was defined using the participant-recalled weight at age 25 years (kg) and the measured height at baseline (m).

Random plasma glucose (RPG) level was measured on-site using the SureStep Plus System (Johnson & Johnson, California, US). Participants with RPG levels ≥7.8 and <11.1 mmol/L were invited to have a fasting plasma glucose (FPG) test the following day. RPG data were missing in a small number (8,156, or 1.6%) of participants, owing to a delay in the availability of on-site test results in certain regions. Previously diagnosed diabetes was defined on the basis of self-reported history of diabetes. Screen-detected diabetes was defined as (i) RPG ≥7.0 mmol/L and time since last eating ≥8 hours, (ii) ≥11.1 mmol/L with time since last eating <8 hours, or (iii) an FPG ≥7.0 mmol/L on subsequent testing among participants without previously diagnosed diabetes [11]. Diabetes was defined as having either previously diagnosed diabetes or screen-detected diabetes.

Details of physical activity measurements have been described elsewhere [12]. The physical activity questionnaire was adapted from validated questionnaires (the Shanghai Women’s Health Study [13] and the European Prospective Investigation into Cancer and Nutrition [14]) and modified after a pilot study. However, these questions had not been validated in CKB separately. The information on usual type and duration of occupational and nonoccupational activities (transportation, housework, and leisure-time exercise) during the past year was collected to calculate metabolic equivalent of task (MET) hours per day (MET-h/day), which was the product of the number of hours spent per day participating in each activity and the MET score for that activity. METs from the 2011 update of a major compendium of physical activities were used [15].

Individuals were classified by smoking status as never, occasional, former regular, or current regular smokers [16]. Never smokers were defined as individuals who reported not smoking at baseline and had smoked <100 cigarettes (or equivalent) in their lifetime. Former regular smokers were defined as individuals who had smoked ≥100 cigarettes (or equivalent) but had quit smoking by choice for ≥6 months before baseline. Occasional smokers were defined as individuals who did not meet the criteria for never smokers and who had not stopped smoking completely for ≥6 months before baseline. Current regular smokers were defined as individuals who reported having smoked ≥1 cigarettes (or equivalent) daily for at least 6 months at baseline. Approximately 50% of former regular smokers stopped smoking because of physical illness that they already had, and they were combined with current regular smokers in the analyses. Among current regular smokers, the amount of smoking was categorised as <20, 20–24, and ≥25 cigarettes (or equivalent) per day. To validate exposure to smoking, exhaled carbon monoxide was measured (CareFusion MicroCO meter, UK) [10]. The validation study showed much higher levels of exhaled carbon monoxide in current regular smokers than in never and former regular smokers, and—among current regular smokers—there were higher levels among those who smoked more and inhaled more deeply [17].

The information on alcohol drinking included drinking frequency, age at which drinking began, and the type and amount consumed on a typical drinking day [18]. The total amount of pure alcohol consumed in grams (g) was estimated based on the beverage type and volume drunk, assuming the following alcohol content by volume (v/v): beer 4%, grape wine 12%, rice wine 15%, weak spirits 38%, and strong spirits 53%. Participants were allowed to report only 1 beverage type for a typical drinking day (i.e., the type of beverage drunk habitually). Drinking categories were abstainers, occasional, reduced-intake, ex-weekly, and weekly drinkers, and—among weekly drinkers—usual consumption categories were <140, 140 to <420, and ≥420 g of alcohol per week [18]. Abstainers were defined as individuals who had never or almost never consumed alcohol in the past year and had not drunk weekly in the past. Occasional drinkers were defined as individuals who in the past year had consumed alcohol occasionally, during certain seasons, or monthly but less than weekly, and had not drunk weekly in the past. Reduced-intake drinkers were individuals who in the past year had consumed alcohol occasionally, during certain seasons, or monthly but less than weekly, but had drunk weekly in the past. Ex-weekly drinkers were individuals who had drunk weekly in the past but had never or almost never consumed alcohol in the past year. Weekly drinkers were individuals who usually drank at least once a week during the past year. Among weekly drinkers, heavy drinking episodes were defined as alcohol consumption of ≥60 g on one occasion for men and ≥40 g for women. The questions on alcohol drinking had not been validated in CKB.

### Follow-up for and ascertainment of disease cases

Each participant had their vital status tracked periodically through China Centre for Disease Control and Prevention (CDC)’s Disease Surveillance Points (DSP) system and national health insurance system (which covers over 98% of our participants, capturing all episodes of hospitalisation, for reimbursement purposes), supplemented by regular checks against local residential and administrative records and by annual active confirmation through street committees or village administrators [19]. In addition, when a patient experienced an episode of major disease and hospitalisation, this was collected through linkage, via each participant’s unique national identification number, with disease registries (for cancer, ischaemic heart disease [IHD], stroke, and diabetes) and national health insurance claims databases. Disease events were coded using International Classification of Diseases, 10th Revision (ICD-10) by trained DSP staff (for death) or medical professionals (for hospitalised events) who were blinded to baseline information [10]. Acute pancreatitis was defined as ICD-10 code K85 and other diseases of the pancreas as K86 (S1 Table). The health insurance data have been shown to be of good quality for other diagnosis codes (e.g., cancer, stroke, IHD, diabetes), but the diagnosis code of pancreatitis has not been validated. The disease standardisation process is described in S1 Text. Only the first event of pancreatitis for each individual was included in the analyses. By 1 January 2015 (censoring date for the present analysis), 1,082 participants (0.2%) had developed acute pancreatitis (ICD-10 K85), 688 (0.1%) had developed other diseases of the pancreas (ICD-10 K86), 30,582 (6%) had died (including 360 due to acute pancreatitis), and 3,898 (0.8%) were lost to follow-up. Participants who died or were lost to follow-up were censored at the last day known to be alive.

### Statistical analysis

We excluded individuals with a prior history of cancer (*n* = 2,577), leaving 510,314 individuals for the main analysis. Due to the extremely low prevalence of smoking (2.8% current regular smokers) and regular alcohol drinking (2.1% weekly drinkers) in women, the analyses for smoking and alcohol were restricted to men only (*n* = 209,237). Numbers of participants with missing data were 2 for BMI at baseline, 82,415 (16%) for BMI25, 241 (0.05%) for %BF, and 8,156 (1.6%) for RPG. There were no missing data for other covariates. In all analyses, a complete case approach was used. The results for BMI25 and RPG were unchanged in a sensitivity analysis using multiple imputation (data not shown).

In the analysis of individual risk factors, Cox proportional hazards regression models were used to estimate adjusted hazard ratios (HRs) for pancreatitis, stratified by sex and study area (10 areas) and adjusted for age at baseline, education (4 groups: no formal school, primary school, middle/high school, or college/university), smoking (4 groups: never, occasional, former regular, or current regular), alcohol (5 groups: abstainers, occasional drinkers, reduced-intake drinkers, ex-weekly drinkers, or weekly drinkers), and medication, where appropriate. Alcohol and smoking were also used individually as exposure variables, adjusting for all remaining variables (except sex). Among the medications that have been reported to be associated with risk of pancreatitis [5,6], we selected those collected in baseline questionnaires (aspirin, angiotensin-converting enzyme inhibitors [ACE-Is], beta blockers, statins, diuretics, Ca^++^ antagonists, metformin, and insulin). Each medication was modelled as a dichotomous variable. Further adjustment for dietary variables (fruits, vegetables, preserved vegetables, dairy, soybean, red meat, fish, poultry) had negligible influence on the examined exposure–outcome associations (data not shown). Time since birth was used as the underlying time scale with delayed entry at age at baseline. The proportional hazards assumption was tested for each model using scaled Schoenfeld residuals, and results showed no substantial evidence of violation of the assumption. Throughout the manuscript, we will use the terms risk and hazard interchangeably because risk is a more generic term that encompasses hazard.

For categorical analyses, BMI was used as a categorical variable with 5 categories (<20.0, 20.0 to <22.5, 22.5 to <25.0, 25.0 to <27.0, and ≥27.0 kg/m^2^). Other measures of adiposity were grouped by splitting at quintiles. The association of WC with pancreatitis was also examined using the International Diabetes Federation (IDF) categories for Chinese (≥90 cm in men and ≥80 cm in women) [20]. For height-adjusted weight, standing height was also included in the model as a continuous variable. RPG was categorised into 4 groups ≤4.4 (reference), >4.4 to <5.6, 5.6 to <7.8, and ≥7.8 mmol/L, selected to include the FPG threshold for diabetes [21]. The analysis for RPG was additionally adjusted for fasting time. Physical activity was grouped by splitting at quintiles. To assess the potentially nonlinear relationships between metabolic risk factors and risk of pancreatitis, restricted cubic splines for each metabolic risk factor were calculated using 3 fixed knots at 10%, 50%, and 90% through the total distribution. Likelihood ratio rests indicated no evidence of significant nonlinear associations for adiposity measures or physical activity (*p* = 0.08–0.99), while there was evidence of a significant nonlinear association for RPG (*p* = 0.001 for acute pancreatitis; *p* = 0.008 for other diseases of the pancreas). All adiposity variables were also modelled as continuous variables to estimate HRs per 1-SD increase, while for physical activity HRs per 4 MET-h/day increase were estimated (equivalent to 1 hour of moderate physical activity such as brisk walking [3–4 mph]).

To examine the prognosis of pancreatitis, Cox models with a time-varying exposure were used to estimate adjusted HRs for incidence of pancreatic cancer (ICD-10 C25) and death by diagnosis of pancreatitis during follow-up, adjusting for the same variables as those in the analysis of metabolic risk factors (as non–time-varying variables). Pancreatitis status after baseline was a time-varying explanatory variable, and individuals were considered as exposed from the time of diagnosis. For analyses involving more than 2 categories, all HRs are presented with 95% CIs calculated using ‘floating’ standard errors to facilitate comparisons between any 2 groups rather than just with the reference group [22]. To investigate the joint effects of metabolic risk factors, we grouped each participant into 1 of 4 categories according to number of metabolic risk factors, including obesity (BMI ≥30 kg/m^2^ or WC ≥100 cm), diabetes, physical inactivity (<17.5 MET-h/day [median of total physical activity in all participants]), and history of gallstone disease. The same variables were adjusted for as in the analysis of individual risk factors, and each metabolic factor was weighted equally. Adjusted HRs were reported for individuals with 1, 2, and ≥3 metabolic risk factors compared with those without any metabolic risk factors.

In sensitivity analysis, we repeated the analyses for individual risk factors with the following exclusions: (1) participants who had previously diagnosed gallbladder disease at baseline, (2) participants who had previously diagnosed major gastrointestinal diseases at baseline (gallbladder disease, peptic ulcer, chronic hepatitis, or cirrhosis), and (3) the first 2 years of follow-up. This is because major gastrointestinal diseases may affect adiposity levels (e.g., BMI and WC) and other lifestyle factors and result in reverse causation (in which lower BMI at study baseline was the result, rather than the cause, of the underlying disease). We excluded the first 2 years of follow-up because participants with subclinical or undetected diseases at baseline might be diagnosed with pancreatitis over early years of follow-up, which could result in reverse causation. We used SAS version 9.3 and R version 2.14.2.

## Results

### Baseline characteristics and diagnosis rate of pancreatitis

Among the 510,314 participants, the mean (SD) age at study entry was 51.5 (10.7) years, and 59% were women. At baseline, the mean BMI and WC were 23.7 (SD 3.4) kg/m^2^ and 80.3 (9.8) cm, 6% had diabetes, and 6% had gallbladder disease (Table 1). During approximately 4.3 million person-years of follow-up, 1,079 participants developed acute pancreatitis, and 683 developed other diseases of the pancreas between the ages of 30 and 79 years, with the mean age at diagnosis being 60.5 years (S1 Table). The diagnosis rate was 23.4 per 100,000 person-years for acute pancreatitis and 14.9 per 100,000 person-years for other diseases of the pancreas. The diagnosis rate was higher in rural than in urban areas (acute pancreatitis: 27.9 versus 17.5 per 100,000 person-years; other diseases of the pancreas: 18.6 versus 10.1 per 100,000 person-years) and was similar among men and women (acute pancreatitis: 22.6 versus 23.9 per 100,000 person-years; other diseases of the pancreas: 16.2 versus 14.0 per 100,000 person-years). For acute pancreatitis, the mortality rate was 8.4 per 100,000 person-years since diagnosis.

**Table 1:** Baseline characteristics of participants in CKB.

Variable^1^	All participants (*n* = 510,314)
Age (SD), year	51.5 (10.7)
Female, %	59.0
**Socioeconomic and lifestyle factors**	
Urban region, %	44.1
Middle school and higher, %	43.4
Annual household income ≥35,000 yuan, %	24.7
Current regular smoker, %	
Male	67.6
Female	2.8
Weekly drinker, %	
Male	33.2
Female	2.1
Total physical activity (SD), MET-h/day	21.1 (13.9)
**Blood pressure and anthropometry**	
SBP (SD), mmHg	131.1 (21.3)
RPG (SD), mmol/L	6.1 (2.3)
BMI (SD), kg/m^2^	23.7 (3.4)
BMI25 (SD), kg/m^2^	21.9 (2.6)
WC (SD), cm	80.3 (9.8)
%BF (SD), %	27.9 (8.4)
**Prior disease history, %**	
Previously diagnosed diabetes	3.1
Screen-detected diabetes	2.8
Gallbladder disease	6.0
Family history of diabetes	4.9
Family history of cancer	14.0

^1^Results were adjusted for age, sex, and region (where appropriate).

Numbers of participants with missing data: BMI *n* = 2; %BF *n* = 241; BMI25 *n* = 82,415; RPG *n* = 8,156.

Abbreviations: %BF, body fat percentage; BMI, body mass index; BMI25, BMI at age 25; CKB, China Kadoorie Biobank; MET-h/day, metabolic equivalent of task hours per day; RPG, random plasma glucose; SBP, systolic blood pressure; WC, waist circumference.

### Adiposity and risk of acute pancreatitis

**Fig 1:**
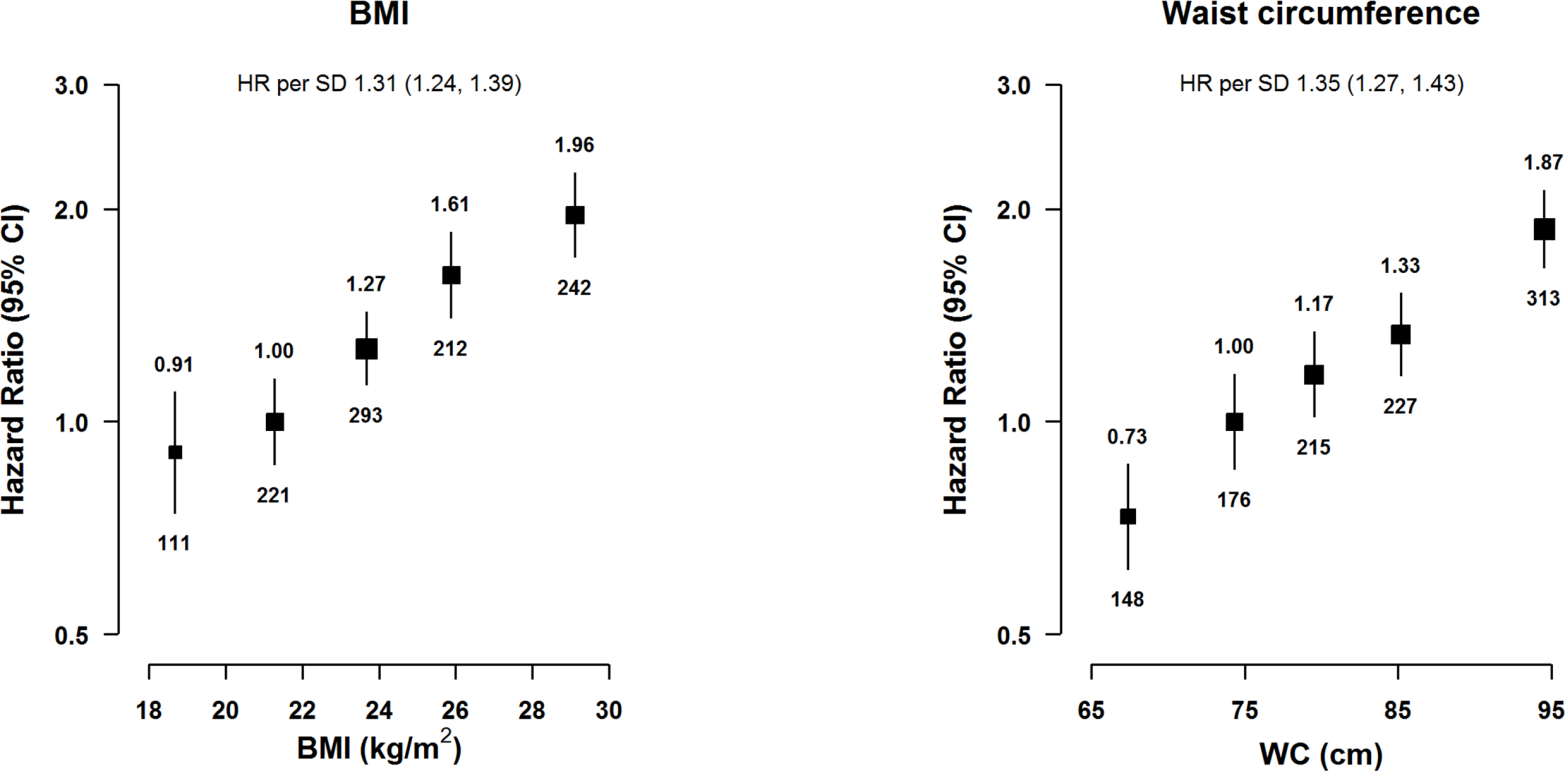
Adjusted HRs for acute pancreatitis by BMI and WC. BMI was classified as <20.0, 20.0 to <22.5 (reference), 22.5 to <25.0, 25.0 to <27.0, and ≥27.0 kg/m^2^. WC was classified by quintiles (<71.7, 71.7 to <77.0 [reference], 77.0 to <82.2, 82.2 to <88.5, and ≥88.5 cm). Model was stratified by sex and region and adjusted for age at baseline, education, smoking, alcohol, and medication (aspirin, ACE-I, beta blockers, statins, diuretics, Ca^++^ antagonists, metformin, and insulin). Time since birth was used as the underlying time scale with delayed entry at age at baseline. HRs were plotted against the mean level in each adiposity group. Log-scale was used for the y-axis. The squares represent HRs, and the vertical lines represent 95% CIs. The area of the squares is inversely proportional to the variance of the log HRs. The numbers above the vertical lines are point estimates for HRs, and the numbers below the lines are numbers of events. BMI was missing in 2 participants. SD was 3.4 kg/m^2^ for BMI and 9.8 cm for WC. ACE-I, angiotensin-converting enzyme inhibitor; BMI, body mass index; HR, hazard ratio; WC, waist circumference.

BMI (a measure of general adiposity) was strongly and positively associated with risk of acute pancreatitis (Fig 1), with an adjusted HR of 1.31 (95% CI 1.24–1.39) per 1-SD (corresponding to 3.4 kg/m^2^)-higher baseline BMI (*p* < 0.001). Similarly, WC (a measure of central adiposity but strongly correlated with BMI: Pearson’s correlation coefficient = 0.83) showed a positive association with risk of acute pancreatitis, with an adjusted HR of 1.35 (1.27–1.43) per 1-SD (equivalent to 9.8 cm)-higher WC (*p* < 0.001). According to the IDF categorisations of adiposity for Chinese individuals, the HR associated with a high WC (≥90 versus <90 cm in men and ≥80 versus <80 cm in women) was 1.87 (1.50–2.34; *p* < 0.001) in men and 1.53 (1.31–1.80; *p* < 0.001) in women (*p* for heterogeneity by sex = 0.15). Other measures of adiposity such as WHR, HC, %BF, and height-adjusted weight were also positively associated with risk of acute pancreatitis (S1 Fig). Additional adjustment for diabetes, a possible mediator on the pathway between adiposity and acute pancreatitis, did not alter the risk estimates (S2 Table). Additional adjustment for WC significantly attenuated the HR for BMI (1.09 [0.97–1.27] per 1-SD-higher BMI; *p* = 0.13, S2 Table), whereas the HR for WC was less influenced by adjustment for BMI (1.25 [1.11–1.41] per 1-SD-higher WC; *p* < 0.001).

### Diabetes, RPG, and risk of acute pancreatitis

**Fig 2:**
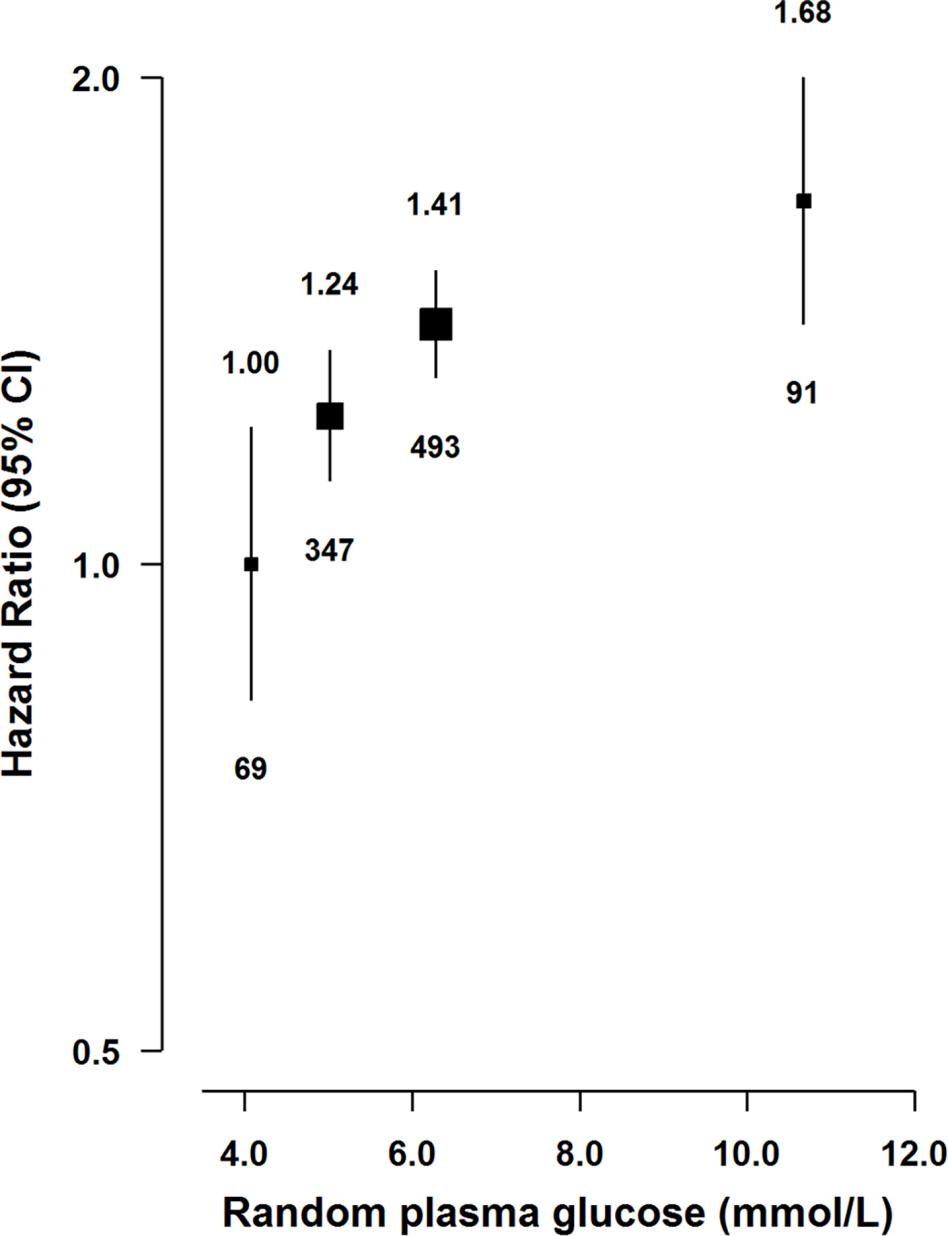
Adjusted HRs for acute pancreatitis by RPG. RPG levels for participants without previously diagnosed diabetes at baseline were classified as ≤4.4 (reference), 4.5 to <5.6, 5.6 to <7.8, and ≥7.8 mmol/L. RPG was missing in 8,156 participants. Model was stratified by sex and region and adjusted for age at baseline, education, smoking, alcohol, medication (aspirin, ACE-I, beta blockers, statins, diuretics, Ca^++^ antagonists, metformin, and insulin), and fasting time. Time since birth was used as the underlying time scale with delayed entry at age at baseline. HRs were plotted against the mean level in each adiposity group. Log-scale was used for the y-axis. The squares represent HRs, and the vertical lines represent 95% CIs. The area of the squares is inversely proportional to the variance of the log HRs. The numbers above the vertical lines are point estimates for HRs, and the numbers below the lines are numbers of events. There was evidence that the association of acute pancreatitis risk with RPG deviated from linearity (*p* = 0.001). The adjusted HR per 1 mmol/L higher RPG was 1.04 (1.02–1.07) (*p* < 0.001). ACE-I, angiotensin-converting enzyme inhibitor; HR, hazard ratio; RPG, random plasma glucose.

Individuals with diabetes had a 34% (HR = 1.34 [1.07–1.69]; *p* = 0.01) higher risk of acute pancreatitis, with similar risks for previously diagnosed and screen-detected diabetes (1.39 [1.17–1.66] and 1.30 [1.03–1.64]; *p =* 0.006 and 0.04, respectively). Additional adjustment for BMI modestly attenuated the association (HR = 1.24 [0.99–1.55] for diabetes; *p* = 0.07). In participants without previously diagnosed diabetes, there was a positive association of RPG with risk of acute pancreatitis (Fig 2), with adjusted HRs of 1.00 (0.83–1.21), 1.24 (1.13–1.35; *p* = 0.018), 1.41 (1.31–1.51; *p* < 0.001), and 1.68 (1.41–1.99; *p* < 0.001) for those with RPG ≤4.4, 4.5 to <5.6, 5.6 to <7.8, and ≥7.8 mmol/L.

### Physical activity, smoking, alcohol, and risk of acute pancreatitis

**Fig 3:**
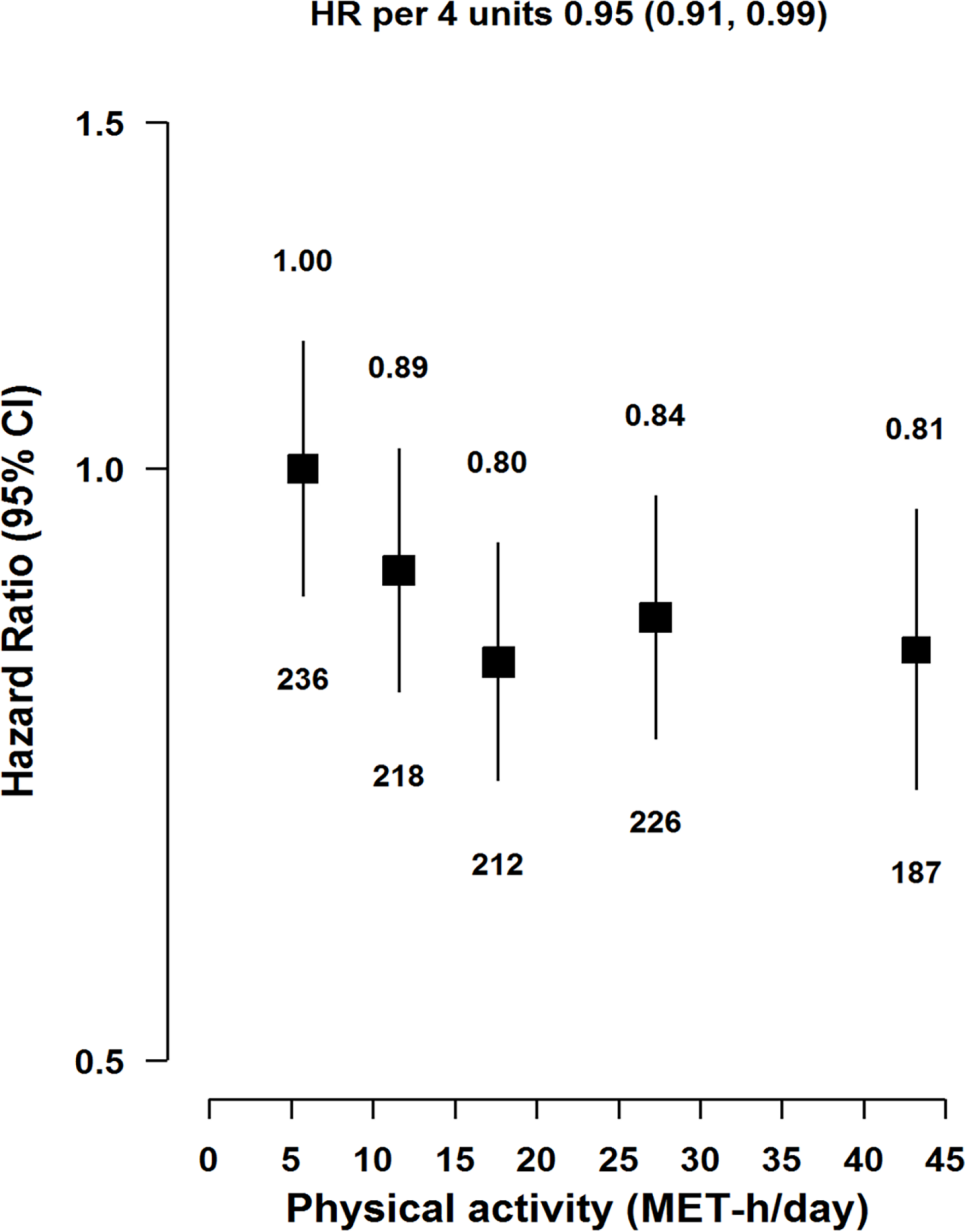
Adjusted HRs for acute pancreatitis by total physical activity. Total physical activity was classified by quintiles (<8.7 [reference], 8.7 to <14.3, 14.3 to <21.9, 21.9 to <33.2, and ≥33.2 MET-h/day). Model was stratified by sex and region and adjusted for age at baseline, education, smoking, alcohol, and medication (aspirin, ACE-I, beta blockers, statins, diuretics, Ca^++^ antagonists, metformin, and insulin). Time since birth was used as the underlying time scale with delayed entry at age at baseline. HRs were plotted against the mean level in each adiposity group. Log-scale was used for the y-axis. The squares represent HRs, and the vertical lines represent 95% CIs. The area of the squares is inversely proportional to the variance of the log HRs. The numbers above the vertical lines are point estimates for HRs, and the numbers below the lines are numbers of events. ACE-I, angiotensin-converting enzyme inhibitor; HR, hazard ratio; MET-h/day, metabolic equivalent of task hours per day.

Physical activity was inversely associated with risk of acute pancreatitis (Fig 3), with each 4 MET-h/day higher usual physical activity associated with an adjusted HR of 0.95 (0.91–0.99; *p* = 0.01). The association was similar for occupational and nonoccupational physical activity (S2 Fig). Compared with never smokers, there were nonsignificant greater risks among occasional smokers (HR = 1.26 [0.93–1.73]; *p* = 0.28) and former regular smokers (1.34 [0.93–1.92]; *p* = 0.21). Compared with never smokers, current regular smokers had 45% (HR = 1.45 [1.28–1.64]; *p* = 0.02) excess risk of acute pancreatitis, with HRs of 1.27 (1.06–1.52), 1.39 (1.16–1.67), and 1.97 (1.59–2.44) for those who smoked <20, 20–24, and ≥25 cigarettes per day, respectively (*p <* 0.001, Table 2). Compared with abstainers, there was a suggestive greater risk among men who reported reduced alcohol intake (HR = 1.40 [0.98–2.01]; *p* = 0.12), while men who drank occasionally, ex-weekly, or weekly were not at a higher risk. Compared with abstainers, men who drank ≥420 g/week had an approximately 50% greater risk (HR = 1.52 [1.11–2.09]; *p* = 0.04), while men who drank <140 or 140 to <420 g/week were not at a higher risk. Among weekly drinkers, heavy drinking episodes were associated with 50% excess risk of acute pancreatitis (HR = 1.50 [1.06–2.11]; *p* = 0.02). Individuals with previously diagnosed gallbladder disease had substantially higher risk of acute pancreatitis (HR = 2.42 [2.03–2.88]; *p* < 0.001).

**Table 2:** Adjusted HRs for acute pancreatitis by smoking status and alcohol consumption among men^1^.

Variable	Number of events	Rate per 100,000 PYs	HR (95% CI)	*p*-Value
**Smoking category**				
Never smokers	49	18.4	1.00 (0.75–1.33)	–
Occasional smokers	40	18.9	1.26 (0.93–1.73)	0.28
Former regular smokers^2^	30	24.3	1.34 (0.93–1.92)	0.21
Current regular smokers	302	24.1	1.45 (1.28–1.64)	0.02
**Cigarette equivalents/day**				
Never smokers	49	18.7	1.00 (0.75–1.34)	–
<20 cigarettes/day	106	20.4	1.27 (1.06–1.52)	0.16
20–24 cigarettes/day	112	22.7	1.39 (1.16–1.67)	0.06
≥25 cigarettes/day	84	35.0	1.97 (1.59–2.44)	<0.001
*p*-Value for trend^3^			<0.001	
**Drinking category**				
Abstainers	84	22.8	1.00 (0.80–1.26)	–
Occasional	136	19.2	1.01 (0.85–1.20)	0.95
Reduced intake	30	33.3	1.40 (0.98–2.01)	0.12
Ex-weekly	19	29.4	1.05 (0.67–1.67)	0.84
Weekly	152	24.2	1.00 (0.85–1.19)	0.98
**Weekly intake (g)**				
Abstainers	84	23.0	1.00 (0.77–1.30)	–
<140	57	25.2	1.02 (0.78–1.34)	0.92
140 to <420	51	17.5	0.73 (0.55–0.96)	0.11
≥420	44	37.6	1.52 (1.11–2.09)	0.04
*p*-Value for trend^3^			0.38	
**Heavy drinking episodes**^**4**^				
No	81	20.6	Reference	
Yes	71	30.3	1.50 (1.06–2.11)	0.02

^1^Model was stratified by region and adjusted for age at baseline, education, smoking, alcohol, and medication (aspirin, ACE-I, beta blockers, statins, diuretics, Ca^++^ antagonists, metformin, and insulin), where appropriate. Time since birth was used as the underlying time scale with delayed entry at age at baseline.

^2^The adjusted HRs were 1.43 (0.68–3.01) and 1.13 (0.86–1.49) for former regular smokers who had stopped due to illness and other reasons, compared with never smokers.

^3^*p*-Value was calculated among current regular smokers using a continuous variable for cigarettes per day and among weekly drinkers using a continuous variable for weekly alcohol intake.

^4^Heavy drinking episodes were defined as the consumption of ≥60 g of alcohol on one occasion for men on a weekly basis, among weekly drinkers. The reference group were weekly drinkers who did not report heavy drinking episodes.

Abbreviations: ACE-I, angiotensin-converting enzyme inhibitor; HR, hazard ratio; PY, person-year.

### Joint effects of metabolic risk factors on acute pancreatitis

When analysed jointly in the same model, the HRs were 1.67 (1.46–1.91; *p* < 0.001) for obesity, 1.22 (0.97–1.54; *p* = 0.08) for diabetes, 1.14 (0.97–1.34, *p* = 0.11) for physical inactivity, and 2.35 (1.98–2.80; *p* < 0.001) for gallbladder disease (Table 3). Among all participants, 37%, 43%, 17%, and 3% had 0, 1, 2, and ≥3 metabolic risk factors (i.e., obesity, diabetes, physical inactivity, gallbladder disease), respectively. Compared with participants without any metabolic risk factors, the adjusted HRs were 1.61 (1.47–1.76), 2.36 (2.01–2.78), and 3.41 (2.46–4.72) for participants with 1, 2, and ≥3 metabolic risk factors, respectively (*p* < 0.001, Table 3).

**Table 3:** Adjusted HRs for acute pancreatitis by metabolic risk factors^1^.

Metabolic risk factors	Number of events	Rates per 100,000 PYs	HR (95% CI)^2^	*p*-Value
**Individual factors**				
Obesity	367	32.6	1.67 (1.46–1.91)	<0.001
Diabetes	86	33.4	1.22 (0.97–1.54)	0.08
Physical inactivity	575	25.3	1.14 (0.97–1.34)	0.11
Gallbladder disease	157	56.8	2.35 (1.98–2.80)	<0.001
**Number of factors**				
0	474	17.2	1.00 (0.90–1.11)	–
1	406	22.9	1.61 (1.47–1.76)	<0.001
2	160	33.6	2.36 (2.01–2.78)	<0.001
≥3	39	52.0	3.41 (2.46–4.72)	<0.001
*p*-Value for trend^3^				<0.001

^1^Obesity was defined as having BMI ≥30 kg/m^2^ or WC ≥100 cm; physical inactivity was defined as <17.5 MET-h/day (median of total physical activity in all participants).

^2^Model was stratified by sex and region and adjusted for age at baseline, education, smoking, alcohol, medication (aspirin, ACE-I, beta blockers, statins, diuretics, Ca^++^ antagonists, metformin, and insulin), and the 3 other metabolic risk factors. Time since birth was used as the underlying time scale with delayed entry at age at baseline.

^3^*p*-Value was calculated treating the number of metabolic factors as a numeric variable.

Abbreviations: ACE-I, angiotensin-converting enzyme inhibitor; BMI, body mass index; HR, hazard ratio; MET-h/day, metabolic equivalent of task hours per day; PY, person-year; WC, waist circumference.

### Acute pancreatitis and risk of death and pancreatic cancer

After diagnosis, individuals with acute pancreatitis had an 8-fold higher risk of pancreatic cancer compared with those without a pancreatitis diagnosis (HR = 8.26 [3.42–19.98]; *p* < 0.001, Table 4). The estimates were similar when excluding pancreatic cancer occurring within 1 or 2 years after pancreatitis diagnosis (Table 4). In addition, individuals with pancreatitis had a higher risk of all-cause mortality (HR = 1.53 [1.17–2.01]; *p* = 0.002, Table 4).

**Table 4:** Adjusted HRs for deaths and major disease outcomes following acute pancreatitis^1^.

Outcome	Number of cases^2^	HR (95% CI)	*p*-Value
**All**			
Pancreatic cancer	13/1,066	8.26 (3.42–19.98)	<0.001
Any death	89/990	1.53 (1.17–2.01)	0.002
**Lag 1 year**^**3**^			
Pancreatic cancer	4/1,057	6.69 (2.15–20.87)	0.001
Any death	44/945	1.51 (1.10–2.08)	0.01
**Lag 2 years**^**3**^			
Pancreatic cancer	3/1,056	9.99 (3.20–31.16)	<0.001
Any death	26/927	1.19 (0.78–1.83)	0.42

^**1**^Model was stratified by sex and region and adjusted for age at baseline, education, smoking, alcohol, and medication (aspirin, ACE-I, beta blockers, statins, diuretics, Ca^++^ antagonists, metformin, and insulin). Time since birth was used as the underlying time scale with delayed entry at age at baseline.

^2^Number of pancreatitis cases that had or had not developed major disease outcomes.

^3^Pancreatitis cases were counted as exposed 1 or 2 years after a diagnosis of pancreatitis.

Abbreviations: ACE-I, angiotensin-converting enzyme inhibitor; HR, hazard ratio.

### Sensitivity analysis

For all risk factors investigated, we found similar associations after excluding participants with prior history of gallbladder disease at baseline, participants with prior history of major gastrointestinal diseases (gallbladder disease, peptic ulcer, chronic hepatitis, or cirrhosis), or when excluding the first 2 years of follow-up (S3 Table). Associations for other diseases of the pancreas are reported in S2 Text (S4–S6 Tables, S2 Fig, S3 Fig).

## Discussion

To our knowledge, this is the first large prospective study in China and one of the largest prospective cohort studies worldwide examining risk factors for acute pancreatitis captured mainly through linkage to hospital records. We found clear evidence that several major modifiable metabolic and lifestyle risk factors were associated with higher risk of acute pancreatitis. For smoking and heavy alcohol drinking, our findings in Chinese adults were generally consistent with those previously reported in Western populations for acute pancreatitis. For metabolic risk factors, our findings for adiposity and diabetes were in agreement with previous studies on acute pancreatitis. Moreover, we showed inverse associations of physical activity with acute pancreatitis risk. Individuals with a diagnosis of acute pancreatitis had higher risks of pancreatic cancer and all-cause mortality.

Three prospective cohort studies in Western populations have previously examined the association of self-reported BMI with risk of acute pancreatitis [23–25]. A Swedish cohort involving 179 acute pancreatitis cases reported a nonsignificant positive trend between BMI and risk of acute pancreatitis [23], while a United States cohort of elderly women with 660 cases of acute or chronic pancreatitis reported a positive association of BMI with acute pancreatitis [24]. CKB included over 1,000 cases of acute pancreatitis and used objectively measured rather than self-reported BMI and showed a positive dose–response association of BMI with risk of acute pancreatitis, stronger than previous studies in Western populations (S7 Table) [23–25]. Moreover, the associations of BMI with acute pancreatitis persisted after excluding the first 2 years of follow-up. To date, one prospective study (68,158 participants and 424 cases) has investigated the association of both BMI and WC with acute pancreatitis [25]. While they reported a null association of self-reported BMI with risk of acute pancreatitis, they found a positive association of self-reported WC, similar to the estimate in CKB. Consistent with this study, we showed that WC was more strongly related to acute pancreatitis than BMI when both were included in the model, suggesting that central adiposity might be more important than general adiposity in the aetiology of acute pancreatitis.

To date, the majority of studies examining the association of diabetes and acute pancreatitis have relied on record linkage [26–31] (i.e., not purpose-built prospective cohort studies), and the only prospective cohort study (660 cases of acute or chronic pancreatitis in elderly women) reported null associations for acute pancreatitis (S7 Table) [24]. Our finding that participants with diabetes had an approximately 30% higher risk of acute pancreatitis was consistent with 5 previous record linkage studies that included 6,524 acute pancreatitis cases [26–31]. We also observed a positive association of RPG with acute pancreatitis among those without a prior diagnosis of diabetes. Although diabetes may be both a consequence and a cause of pancreatitis [32], our finding that the association persisted when excluding the first 2 years of follow-up provides some evidence that the association may not be entirely driven by reverse causality.

In addition to adiposity and diabetes, physical inactivity is related to insulin resistance syndrome, which has been hypothesised as a possible mechanism for diseases of the pancreas [33]. In CKB, we showed that higher physical activity was associated with lower risk of acute pancreatitis, with similar associations for occupational and nonoccupational physical activity. While the risk estimates for nonoccupational physical activity and acute pancreatitis were not statistically significant, this may be due to the narrow range covered.

Several prospective studies in Western populations have consistently shown that smokers were at significantly higher risk of pancreatitis, with similar excess risks for acute and other diseases of the pancreas. However, the reported estimates varied greatly between different studies, ranging from 1.1 to 3.3 for current smokers and 1.1 to 2.3 for ex-smokers [23,24,34–36]. Our risk estimate for current smoking was weaker than most previous studies of Western populations, probably because a relatively low proportion of adult men in China have smoked cigarettes persistently since early adulthood [37]. A meta-analysis of 2 prospective cohorts and 5 case-control studies of 3,618 cases of pancreatitis (2,075 acute and 1,298 chronic pancreatitis) has reported positive, linear associations of alcohol drinking with risks of acute pancreatitis in men and chronic pancreatitis in both sexes [38]. Most previous prospective studies in North America and Europe have shown reasonably consistently that heavy alcohol drinking (i.e., ≥420 g/week) was associated with 60% to 200% higher risk of pancreatitis [24,39,40], again with similar excess risks by pancreatitis subtypes [40]. In China, most people tended to drink alcohol with meals [18]. In CKB, we showed 52% higher risk of acute pancreatitis associated with heavy alcohol drinking, which was weaker than previous studies. In addition, our study suggested that men who reported reduced alcohol intake had a nonsignificant 40% higher risk of acute pancreatitis, which may be partly due to reverse causation.

Of all the proposed aetiological factors of pancreatitis, gallstones are the most common cause, with the proportion of pancreatitis cases due to gallstones ranging from 10% to 60% in Western countries [1]. Although there is no prospective study in China, nationwide retrospective surveys estimated that gallbladder disease accounted for 59% of acute and 34% of chronic pancreatitis cases [41,42]. Our finding for acute pancreatitis in CKB was qualitatively consistent with 3 record linkage studies in Western populations and nationwide surveys [27,29,30,41–43], in which previously diagnosed gallbladder disease was associated with a higher risk of acute pancreatitis. However, studies in Western populations have suggested that gallstones are not a major cause of chronic pancreatitis [1] and that gallstones may not be a risk factor for recurrent or chronic pancreatitis among patients with acute pancreatitis [44]. Although aetiologies of pancreatitis may differ between China and Western countries, the positive association between gallbladder disease and chronic pancreatitis in our study warrants further investigation in other studies of East Asian populations.

Although CKB was not designed to be nationally representative and the participation rate was relatively low, this ought not to reduce the generalisability of our findings to the Chinese adult population, especially given the size and diversity of the CKB and the minimal loss to follow-up. The strengths of the CKB include its prospective design, the large and diverse study population with complete follow-up, and the ability to assess a range of risk factors for pancreatitis, including lifestyle risk factors, anthropometry, and past medical history. This study also has limitations. First, the ICD-10 diagnoses of pancreatitis were not validated against a gold standard, but for other disease outcomes, we have found good to excellent quality of case ascertainment using the health insurance data [45]. Second, as an observational study, residual confounding may still be present, especially that related to smoking and diet. Third, we did not collect information on previously diagnosed pancreatitis at study baseline, and therefore participants might have had pancreatitis episodes before baseline, meaning that the associations could be influenced by reverse causation (i.e., patients with pancreatitis may modify their behaviours or develop diabetes as a result of the disease). However, we found similar associations when excluding major gastrointestinal diseases at baseline, which might be expected to pick up nonspecific symptoms from some forms of pancreatitis (S3 Table). Fourth, for physical activity, smoking, and alcohol, we used self-reported questionnaire data, so misclassification may exist, which may bias the risk estimates.

In summary, among Chinese adults, several metabolic (adiposity, diabetes, physical inactivity, gallbladder disease) and lifestyle (smoking and heavy alcohol drinking among men) factors were strongly associated with higher risk of acute pancreatitis. The strong link between pancreatitis and pancreatic cancer, as well as their shared risk factors (i.e., smoking, heavy drinking, adiposity, diabetes), provide valuable insights into disease aetiology and warrant future studies. Future studies might investigate potential mediators of these associations such as blood-based metabolomics or proteomics, which might be amenable to therapeutic modification and provide new opportunities to treat and prevent acute pancreatitis as well as inform early diagnosis of pancreatic cancer.

## Supporting information

S1 STROBE Checklist (DOCX)

S1 Text Disease standardisation. (DOCX)

S2 Text Other diseases of the pancreas. (DOCX)

S1 Table Distributions of diseases of the pancreas. (DOCX)

S2 Table Adjusted HRs for acute pancreatitis by adiposity. HR, hazard ratio.
(DOCX)

S3 Table Adjusted HRs for acute pancreatitis associated with metabolic and lifestyle risk factors with different exclusions. HR, hazard ratio.
(DOCX)

S4 Table Adjusted HRs for pancreatitis subtypes associated with metabolic and lifestyle risk factors. HR, hazard ratio.
(DOCX)

S5 Table Adjusted HRs for other diseases of the pancreas by smoking status and alcohol consumption among men. HR, hazard ratio.
(DOCX)

S6 Table Adjusted HRs for deaths and major disease outcomes following other diseases of the pancreas. HR, hazard ratio.
(DOCX)

S7 Table Selected key characteristics of published prospective studies of risk factors for pancreatitis. (DOCX)

S1 Fig Adjusted HRs for acute pancreatitis by adiposity measures. HR, hazard ratio.
(TIF)

S2 Fig Adjusted HRs for pancreatitis subtypes by quintiles of physical activity. HR, hazard ratio.
(TIF)

S3 Fig Adjusted HRs for pancreatitis subtypes by RPG. HR, hazard ratio; RPG, random plasma glucose.
(TIF)

## Abbreviations

%BF: body fat percentage
ACE-I: angiotensin-converting enzyme inhibitor
BMI: body mass index
BMI25: BMI at age 25
China CDC: China Centre for Disease Control and Prevention
CKB: China Kadoorie Biobank
DSP: Disease Surveillance Points
FPG: fasting plasma glucose
HC: hip circumference
HR: hazard ratio
ICD-10: International Classification of Diseases, 10th Revision
IDF: International Diabetes Federation
IHD: ischaemic heart disease
MET-h/day: metabolic equivalent of task hours per day
RPG: random plasma glucose
SBP: systolic blood pressure
WC: waist circumference
WHR: waist-to-hip ratio
