# Supplementary material for: Metabolic and lifestyle risk factors for acute pancreatitis in Chinese adults: A prospective cohort study of 0.5 million people
Source: PLoS Med. 2018 Aug 1;15(8):e1002618. doi: 10.1371/journal.pmed.1002618 (PMC6070164; doi:10.1371/journal.pmed.1002618)
Supplement: S1 Text — (DOCX) [file pmed.1002618.s002.docx]

# ****S1 Text. Disease standardisation****

Electronic health records are standardised to ICD10. Mandarin-speaking clinicians assign ICD10 codes to any free format disease description(s) using a recently developed bespoke standardisation tool. The ICD10 diagnoses are then verified by another clinician. A set of diagnoses are produced for each participant, showing which diseases they have developed and when. These data are then imported and linked into our research database in Oxford.
