# Supplementary material for: Metabolic and lifestyle risk factors for acute pancreatitis in Chinese adults: A prospective cohort study of 0.5 million people
Source: PLoS Med. 2018 Aug 1;15(8):e1002618. doi: 10.1371/journal.pmed.1002618 (PMC6070164; doi:10.1371/journal.pmed.1002618)
Supplement: S2 Text — (DOCX) [file pmed.1002618.s003.docx]

# S2 Text. Other diseases of the pancreas

For other diseases of the pancreas, overall we observed similar patterns of risk factors as acute pancreatitis (**S4 Table, S5 Table, S2 Fig, S3 Fig**). However, the associations for alcohol intake were overall weak and non-significant, except that men who reported reduced alcohol intake had a higher risk compared with abstainers (HR=1.69 [1.17-2.45], *p*=0.02). For chronic pancreatitis, although similar patterns were observed for diabetes, smoking, heavy drinking, and gallbladder disease, the associations were non-significant, possibly due to the small number of cases (**S4 Table, S2 Fig, S3 Fig**). For adiposity, there was an inverse trend between BMI and risk of chronic pancreatitis, and a null association for WC (**S4 Table**). After diagnosis, individuals with other diseases of the pancreas were also at increased risks of developing pancreatic cancer and death (**S6 Table**), while the number of chronic pancreatitis cases was too small to permit such an analysis.
