# Supplementary material for: Metabolic and lifestyle risk factors for acute pancreatitis in Chinese adults: A prospective cohort study of 0.5 million people
Source: PLoS Med. 2018 Aug 1;15(8):e1002618. doi: 10.1371/journal.pmed.1002618 (PMC6070164; doi:10.1371/journal.pmed.1002618)
Supplement: S1 Table — (DOCX) [file pmed.1002618.s004.docx]

# ****S1 Table. Distributions of diseases of the pancreas****

| **ICD10 code** | **ICD-10 definition** | **No. cases** |
| --- | --- | --- |
|  |  |  |
| **Acute pancreatitis** | | **1079** |
| K85.1 | Biliary acute pancreatitis | 11 |
| K85.8 | Other acute pancreatitis | 5 |
| K85.9 | Acute pancreatitis, unspecified | 1063 |
|  |  |  |
| **Other diseases of the pancreas** | | **683** |
| K86.1 | Other chronic pancreatitis | 113 |
| K86.2 | Cyst of pancreas | 12 |
| K86.3 | Pseudocyst of pancreas | 7 |
| K86.8 | Other specified diseases of pancreas | 7 |
| K86.9^1^ | Disease of pancreas, unspecified | 544 |

^1^ Of the 356 cases whose medical records could be retrieved, 42.7% (152/356) of patients had either concomitant

or prior diagnoses of gallbladder disease.
