# Supplementary material for: Metabolic and lifestyle risk factors for acute pancreatitis in Chinese adults: A prospective cohort study of 0.5 million people
Source: PLoS Med. 2018 Aug 1;15(8):e1002618. doi: 10.1371/journal.pmed.1002618 (PMC6070164; doi:10.1371/journal.pmed.1002618)
Supplement: S2 Table — HR, hazard ratio. (DOCX) [file pmed.1002618.s005.docx]

# S2 Table. Adjusted HRs for acute pancreatitis by adiposity

| **Variable** | **No. events** | **Rate**  **per 100,000 PY** | **HR1 (95% CI)^1^** | ***p*-value** | **HR2 (95% CI)^2^** | ***p*-value** |
| --- | --- | --- | --- | --- | --- | --- |
| **BMI (kg/m^2^)** |  |  |  |  |  |  |
| <20.0 | 111 | 19.3 | 0.91 (0.75, 1.10) | 0.39 | 0.91 (0.75, 1.10) | 0.41 |
| 20.0 <22.5 | 221 | 18.6 | 1.00 (0.87, 1.14) | – | 1.00 (0.87, 1.14) | – |
| 22.5 to <25.0 | 293 | 22.3 | 1.27 (1.13, 1.42) | 0.01 | 1.27 (1.13, 1.42) | 0.01 |
| 25.0 to <27.0 | 212 | 27.3 | 1.61 (1.41, 1.85) | <0.001 | 1.60 (1.40, 1.83) | <0.001 |
| ≥27.0 | 242 | 32.2 | 1.96 (1.72, 2.24) | <0.001 | 1.94 (1.70, 2.22) | <0.001 |
| per 1-SD | – | – | 1.31 (1.24, 1.39) | <0.001 | 1.31 (1.23, 1.39) | <0.001 |
| per 1-SD^3^ | – | – | 1.09 (0.97, 1.27) | 0.13 | – | 0.10 |
|  |  |  |  |  |  |  |
| **WC (cm)** |  |  |  |  |  |  |
| <71.7 | 148 | 16.0 | 0.73 (0.62, 0.87) | 0.01 | 0.74 (0.62, 0.87) | 0.01 |
| 71.7 to <77.0 | 176 | 19,8 | 1.00 (0.86, 1.16) | – | 1.00 (0.86, 1.16) | – |
| 77.0 to <82.2 | 215 | 22.5 | 1.17 (1.02, 1.33) | 0.13 | 1.16 (1.02, 1.33) | 0.14 |
| 82.2 to <88.5 | 227 | 24.6 | 1.33 (1.17, 1.51) | 0.01 | 1.32 (1.16, 1.50) | 0.01 |
| ≥88.5 | 313 | 34.1 | 1.87 (1.66, 2.11) | <0.001 | 1.85 (1.64, 2.09) | <0.001 |
| per 1-SD | – | – | 1.35 (1.27, 1.43) | <0.001 | 1.34 (1.26, 1.43) | <0.001 |
| per 1-SD^3^ | – | – | 1.25 (1.11, 1.41) | <0.001 | – | <0.001 |

^1^ Model was stratified by sex and region, and adjusted for age at baseline, education, smoking, alcohol, and medication (aspirin, ACE-I, beta-blockers, statins, diuretics, Ca^++^ antagonists, metformin, and insulin). Time since birth was used as the underlying time scale with delayed entry at age at baseline. SD was 3.4 kg/m^2^ for BMI and 9.8 cm for WC.

^2^ Additionally adjusted for diabetes.

^3^ Additionally adjusted for WC (for BMI) and BMI (for WC).

Abbreviations: ACE-I, angiotensin-converting enzyme inhibitor; BMI, body mass index; HR, hazard ratio; WC, waist circumference.
