# Supplementary material for: Metabolic and lifestyle risk factors for acute pancreatitis in Chinese adults: A prospective cohort study of 0.5 million people
Source: PLoS Med. 2018 Aug 1;15(8):e1002618. doi: 10.1371/journal.pmed.1002618 (PMC6070164; doi:10.1371/journal.pmed.1002618)
Supplement: S3 Table — HR, hazard ratio. (DOCX) [file pmed.1002618.s006.docx]

# S3 Table. Adjusted HRs for acute pancreatitis associated with metabolic and lifestyle risk factors with different exclusions

| **Variable** | **No. events** | **HR (95% CI)**^1^ | ***p*-value** |
| --- | --- | --- | --- |
| ***Excluding baseline gallbladder diseases*** | |  |  |
| **Adiposity** |  |  |  |
| BMI per 1-SD | 922 | 1.35 (1.26, 1.44) | <0.001 |
| WC per 1-SD | 922 | 1.37 (1.28, 1.47) | <0.001 |
| **Total diabetes** | 64 | 1.21 (0.93, 1.57) | 0.16 |
| **Physical activity per 4 MET-h/day** | 922 | 0.96 (0.91, 1.00) | 0.07 |
| **Smoking^2^** |  |  |  |
| Never smokers | 44 | 1.00 (0.74, 1.36) | – |
| Occasional smokers | 36 | 1.24 (0.89, 1.73) | 0.32 |
| Former regular smokers | 28 | 1.45 (0.99, 2.10) | 0.38 |
| Current regular smokers | 270 | 1.44 (1.26, 1.64) | 0.02 |
| **Heavy drinking episodes^3^** | 60 | 1.46 (1.02, 2.10) | 0.04 |
|  | |  |  |
| ***Excluding baseline gastrointestinal diseases*** | |  |  |
| **Adiposity** |  |  |  |
| BMI per 1-SD | 870 | 1.35 (1.26, 1.44) | <0.001 |
| WC per 1-SD | 870 | 1.38 (1.29, 1.48) | <0.001 |
| **Total diabetes** | 64 | 1.28 (0.98, 1.66) | 0.07 |
| **Physical activity per 4 MET-h/day** | 870 | 0.96 (0.92, 1.01) | 0.14 |
| **Smoking^2^** |  |  |  |
| Never smokers | 41 | 1.00 (0.73, 1.37) | – |
| Occasional smokers | 34 | 1.28 (0.91, 1.79) | 0.28 |
| Former regular smokers | 27 | 1.47 (1.01, 2.15) | 0.57 |
| Current regular smokers | 246 | 1.42 (1.23, 1.62) | 0.02 |
| **Heavy drinking episodes^3^** | 58 | 1.47 (1.02, 2.11) | 0.04 |
|  | |  |  |
| ***Excluding the first two years of follow-up*** | |  |  |
| **Adiposity** |  |  |  |
| BMI per 1-SD | 962 | 1.28 (1.20, 1.37) | <0.001 |
| WC per 1-SD | 962 | 1.32 (1.24, 1.41) | <0.001 |
| **Total diabetes** | 68 | 1.28 (0.99, 1.64) | 0.11 |
| **Physical activity per 4 MET-h/day** | 962 | 0.96 (0.91, 1.00) | 0.06 |
| **Smoking^2^** |  |  |  |
| Never smokers | 36 | 1.00 (0.72, 1.40) | – |
| Occasional smokers | 33 | 1.43 (1.02, 2.02) | 0.13 |
| Former regular smokers | 28 | 1.72 (1.18, 2.50) | 0.08 |
| Current regular smokers | 261 | 1.69 (1.48, 1.93) | <0.001 |
| **Heavy drinking episodes^3^** | 64 | 1.63 (1.12, 2.36) | 0.01 |

^1^ Model was stratified by sex and region, and adjusted for age at baseline, education, smoking, alcohol, and medication (aspirin, ACE-I, beta-blockers, statins, diuretics, Ca^++^ antagonists, metformin, and insulin), where appropriate. Time since birth was used as the underlying time scale with delayed entry at age at baseline.

^2^ The analysis was restricted to male participants.

^3^ The analysis was restricted to male participants. Heavy drinking episodes were defined as the consumption of ≥60 g of alcohol on one occasion for men on a weekly basis, among weekly drinkers. The reference group were weekly-drinkers who did not report heavy drinking episodes.

Abbreviations: ACE-I, angiotensin-converting enzyme inhibitor; BMI, body mass index; HR, hazard ratio; MET-h/day, metabolic equivalent of task hours per day; WC, waist circumference.
