# Supplementary material for: Metabolic and lifestyle risk factors for acute pancreatitis in Chinese adults: A prospective cohort study of 0.5 million people
Source: PLoS Med. 2018 Aug 1;15(8):e1002618. doi: 10.1371/journal.pmed.1002618 (PMC6070164; doi:10.1371/journal.pmed.1002618)
Supplement: S4 Table — HR, hazard ratio. (DOCX) [file pmed.1002618.s007.docx]

# S4 Table. Adjusted HRs for pancreatitis subtypes associated with metabolic and lifestyle risk factors

| **Variable** | **No. events** | | **HR (95% CI)**^1^ | ***p*-value** |
| --- | --- | --- | --- | --- |
| ***Acute pancreatitis*** | | | |  |
| BMI per 1-SD | | 1079 | 1.31 (1.24, 1.39) | <0.001 |
| WC per 1-SD | | 1079 | 1.35 (1.27, 1.43) | <0.001 |
| Diabetes | | 86 | 1.34 (1.07, 1.69) | 0.01 |
| Physical activity per 4 MET-h/day | | 1079 | 0.95 (0.91, 0.99) | 0.03 |
| Gallbladder disease | | 157 | 2.42 (2.03, 2.88) | <0.001 |
| Current regular smoking^2^ | | 302 | 1.45 (1.28, 1.64) | 0.02 |
| Weekly drinking ≥420 g^3^ | | 44 | 1.52 (1.11, 2.09) | 0.04 |
|  | |  |  |  |
| ***Other pancreatitis*** | | | |  |
| BMI per 1-SD | | 683 | 1.24 (1.15, 1.34) | <0.001 |
| WC per 1-SD | | 683 | 1.30 (1.20, 1.40) | <0.001 |
| Diabetes | | 67 | 1.73 (1.33, 2.24) | <0.001 |
| Physical activity per 4 MET-h/day | | 683 | 0.95 (0.90, 0.99) | 0.04 |
| Gallbladder disease | | 112 | 2.63 (2.14, 3.24) | <0.001 |
| Current regular smoking^2^ | | 222 | 1.47 (1.28, 1.70) | 0.04 |
| Weekly drinking ≥420 g^3^ | | 24 | 1.16 (0.76, 1.76) | 0.57 |
|  | |  |  |  |
| ***Chronic pancreatitis*** | | | |  |
| BMI per 1-SD | | 113 | 0.86 (0.70, 1.06) | 0.16 |
| WC per 1-SD | | 113 | 1.00 (0.82, 1.22) | 0.99 |
| Diabetes | | 9 | 1.69 (0.84, 3.40) | 0.14 |
| Physical activity per 4 MET-h/day | | 113 | 0.96 (0.86, 1.08) | 0.50 |
| Gallbladder disease | | 19 | 3.04 (1.83, 5.06) | <0.001 |
| Current regular smoking^2^ | | 38 | 1.60 (0.61, 4.19) | 0.34 |
| Weekly drinking ≥420 g^3^ | | 8 | 2.10 (0.82, 5.34) | 0.12 |

^1^ Model was stratified by sex and region, and adjusted for age at baseline, education, smoking, alcohol, and medication (aspirin, ACE-I, beta-blockers, statins, diuretics, Ca^++^ antagonists, metformin, and insulin), where appropriate. Time since birth was used as the underlying time scale with delayed entry at age at baseline.

^2^ The analysis was restricted to male participants and the reference group was never smoker.

^3^ The analysis was restricted to male participants and the reference group was abstainer.

Abbreviations: ACE-I, angiotensin-converting enzyme inhibitor; BMI, body mass index; HR, hazard ratio; MET-h/day, metabolic equivalent of task hours per day; WC, waist circumference.
