# Supplementary material for: Metabolic and lifestyle risk factors for acute pancreatitis in Chinese adults: A prospective cohort study of 0.5 million people
Source: PLoS Med. 2018 Aug 1;15(8):e1002618. doi: 10.1371/journal.pmed.1002618 (PMC6070164; doi:10.1371/journal.pmed.1002618)
Supplement: S5 Table — HR, hazard ratio. (DOCX) [file pmed.1002618.s008.docx]

# S5 Table. Adjusted HRs for other pancreatitis by smoking status and alcohol consumption among men

| **Variable** | | **No. of events** | | | | **Rate per 100,000 PY** | | | **HR (95% CI)**^1^ | | ***p*-value** | |
| --- | --- | --- | --- | --- | --- | --- | --- | --- | --- | --- | --- | --- |
| **Smoking category** | | |  | | |  | | |  | |  |  |
| Never smokers | | | 33 | | | 12.4 | | | 1.00 (0.70, 1.42) | | – |  |
| Occasional smokers | | | 26 | | | 12.3 | | | 1.17 (0.79, 1.72) | | 0.56 |  |
| Former regular smokers | | | 18 | | | 14.6 | | | 1.09 (0.68, 1.73) | | 0.77 |  |
| Current regular smokers | | | 222 | | | 17.7 | | | 1.47 (1.28, 1.70) | | 0.04 |  |
|  | | |  | | |  | | |  | |  |  |
| **Cigarette equivalents/day** | | |  | | |  | | |  | |  |  |
| Never smokers | | | 33 | | | 12.7 | | | 1.00 (0.70, 1.43) | | – |  |
| <20 cigarettes/day | | | 77 | | | 13.9 | | | 1.13 (0.91, 1.41) | | 0.55 |  |
| 20–24 cigarettes/day | | | 86 | | | 18.2 | | | 1.57 (1.28, 1.94) | | 0.03 |  |
| ≥25 cigarettes/day | | | 59 | | | 23.8 | | | 1.88 (1.45, 2.44) | | 0.01 |  |
| *p-*value for trend^2^ | | |  | | |  | | | *0.01* | |  |  |
|  | | |  | | |  | | |  | |  |  |
| **Drinking category** | | |  | | |  | | |  | |  |  |
| Abstainers | | | 68 | | | 18.5 | | | 1.00 (0.77, 1.29) | | – |  |
| Occasional | | | 96 | | | 12.0 | | | 0.87 (0.69, 1.08) | | 0.41 |  |
| Reduced intake | | | 9 | | | 32.2 | | | 1.69 (1.17, 2.45) | | 0.02 |  |
| Ex-weekly | | | 29 | | | 31.0 | | | 1.30 (0.83, 2.04) | | 0.31 |  |
| Weekly | | | 97 | | | 15.5 | | | 0.85 (0.68, 1.05) | | 0.33 |  |
|  | | | |  | | | |  | | |  | |
| **Weekly intake (gram)** | | | |  | | | |  | | |  | |
| Abstainers | 68 | | | | 18.4 | | 1.00 (0.74, 1.35) | | | – | | |
| <140 | 31 | | | | 13.7 | | 0.76 (0.53, 1.09) | | | 0.24 | | |
| 140 to <420 | 42 | | | | 14.7 | | 0.82 (0.61, 1.11) | | | 0.37 | | |
| ≥420 | 24 | | | | 20.5 | | 1.16 (0.76, 1.76) | | | 0.57 | | |
| *p-*value for trend^2^ |  | | | |  | | *0.95* | | |  | | |
|  |  | | | |  | |  | | |  | | |
| **Heavy drinking episodes^3^** | | | | |  | |  | | |  | | |
| No | 55 | | | | 14.0 | | Reference | | |  | | |
| Yes | 42 | | | | 17.9 | | 1.25 (0.82, 1.91) | | | 0.30 | | |

^1^ Model was stratified by region, and adjusted for age at baseline, education, smoking, alcohol, and medication (aspirin, ACE-I, beta-blockers, statins, diuretics, Ca^++^ antagonists, metformin, and insulin), where appropriate. Time since birth was used as the underlying time scale with delayed entry at age at baseline.

^2^ *P-*value was calculated among current regular smokers using a continuous variable for cigarettes per day and among weekly drinkers using a continuous variable for weekly alcohol intake.

^3^ Heavy drinking episodes were defined as the consumption of ≥60 g of alcohol on one occasion for men on a weekly basis, among weekly drinkers. The reference group were weekly-drinkers who did not report heavy drinking episodes.

Abbreviations: ACE-I, angiotensin-converting enzyme inhibitor; HR, hazard ratio; PY, person-year.
