# Supplementary material for: Metabolic and lifestyle risk factors for acute pancreatitis in Chinese adults: A prospective cohort study of 0.5 million people
Source: PLoS Med. 2018 Aug 1;15(8):e1002618. doi: 10.1371/journal.pmed.1002618 (PMC6070164; doi:10.1371/journal.pmed.1002618)
Supplement: S6 Table — HR, hazard ratio. (DOCX) [file pmed.1002618.s009.docx]

# S6 Table. Adjusted HRs for deaths and major disease outcomes following other pancreatitis^1^

| **Outcome** | **No. cases**^2^ | **HR (95% CI)** | ***p*-value** |
| --- | --- | --- | --- |
| ***All*** |  |  |  |
| Pancreatic cancer | 92/591 | 9.39 (3.88, 22.73) | <0.001 |
| Any death | 17/666 | 1.87 (1.46, 2.40) | <0.001 |
|  |  |  |  |
| ***Lag 1 year^3^*** |  |  |  |
| Pancreatic cancer | 50/549 | 4.79 (1.19, 19.25) | 0.03 |
| Any death | 4/653 | 1.64 (1.22, 2.21) | 0.001 |
|  |  |  |  |
| ***Lag 2 years^3^*** |  |  |  |
| Pancreatic cancer | 37/536 | 6.54 (1.62, 26.29) | 0.008 |
| Any death | 2/651 | 1.55 (1.09, 2.22) | 0.02 |

**^1^** Model was stratified by sex and region, and adjusted for age at baseline, education, smoking, alcohol, and medication (aspirin, ACE-I, beta-blockers, statins, diuretics, Ca^++^ antagonists, metformin, and insulin). Time since birth was used as the underlying time scale with delayed entry at age at baseline.

^2^ Number of pancreatitis cases that had or had not developed major disease outcomes.

^3^ Pancreatitis cases were counted as exposed one or two years after a diagnosis of pancreatitis.

Abbreviations: ACE-I, angiotensin-converting enzyme inhibitor; HR, hazard ratio.
