# Supplementary material for: Metabolic and lifestyle risk factors for acute pancreatitis in Chinese adults: A prospective cohort study of 0.5 million people
Source: PLoS Med. 2018 Aug 1;15(8):e1002618. doi: 10.1371/journal.pmed.1002618 (PMC6070164; doi:10.1371/journal.pmed.1002618)
Supplement: S7 Table — (DOCX) [file pmed.1002618.s010.docx]

# S7 Table. Selected key characteristics of published prospective studies of risk factors for pancreatitis

| **Author** | **Study population** | **No. of participants** | **No. of cases** | **Follow-up** | **Age** | **First AP** | **Risk factor ascertainment** | **Category** | **RR (95% CI)** |
| --- | --- | --- | --- | --- | --- | --- | --- | --- | --- |
| **Adiposity** | | | | | | | | | |
| Lindkvist, 2008, Sweden [23] | The Malmö Preventive Project | 33,346 | 179 | NA | ≥45 | Yes | measured | BMI  <20.0  20.0-24.9  25.0-29.9  ≥30.0 | **AP**  1.02 (0.56, 1.88)  1.00  1.15 (0.83, 1.60)  1.45 (0.85, 2.48) |
| Prizment, 2015, US [24] | Iowa Women’s Health Study | 36,436 | AP 511  CP 149 | NA | ≥65 | No | self-report | BMI  <25.0  25.0-29.9  ≥30.0  BMI  <25.0  25.0-29.9  ≥30.0 | **AP**  1.00  1.29 (1.05, 1.60)  1.35 (1.07, 1.70)  **CP**  1.00  0.67 (0.46, 0.97)  0.59 (0.37, 0.94) |
| Sadr-Azodi, 2013, Sweden [25] | Swedish Mammography Cohort and Cohort of Swedish Men | 68,158 | 424 | 12 yrs | 46-84 | Yes | self-report | BMI  <25.0  25.0-29.9  ≥30.0  WC per 10cm | **AP**  1.00  1.18 (0.92, 1.51)  1.02 (0.68, 1.53)  1.35 (1.19, 1.53) |
| **Diabetes** | | | | | | | | | |
| Prizment, 2015, US [24] | Iowa Women’s Health Study | 36,436 | AP 511  CP 149 | NA |  | No | self-report | diabetes | **AP** 1.01 (0.69, 1.47)  **CP** 1.20 (0.60, 2.39) |
| Gonzalez-Perez, 2010, UK* [26] | The Health Improvement Network database | 85,525 T2DM patients, 200,000 controls | 419 | 4 yrs | mean 60 | No | medical record | diabetes | **AP** 1.79 (1.48, 2.18) |
| Urushihara, 2012, Japan* [27] | hospital administrative database in Japan | 1375 cases, 5469 controls | 1375 | NA | mean: controls 62 DM 67 | No | medical record | diabetes | **AP** 1.86 (1.51, 2.29) |
| Lai, 2011, Taiwan* [28] | Taiwan National Health Insurance database | 19,518 T2DM patients, 78,072 controls | 916 | 3-8 yrs | mean 56.7 | Yes | medical record | diabetes | **AP** 1.89 (1.65, 2.18) |
| Garg, 2010, US* [29] | a large medical and pharmacy claims database | 38,615 diabetes patients, 748,041 controls | 508 | NA | mean 52 | No | medical record | diabetes | **AP** 2.1 (1.7, 2.5) |
| Girman, 2010, UK* [30] | General Practice Research Database | 2,984,755, 5.0% with T2DM | 2735 | NA | mean 47.3 | Yes | medical record | diabetes | **AP** 1.49 (1.31, 1.70) |
| Noel, 2009, US* [31] | US health care claims database | 337,067 T2DM patients, 337,067 controls | 571 | NA | ≥18 | No | medical record | diabetes | **AP**  F 2.59 (2.30, 2.92)  M 3.02 (2.72, 3.35) |
| **Smoking** | | | | | | | | | |
| Prizment, 2015, US [24] | Iowa Women’s Health Study | 36,436 | AP 511  CP 149 | NA | ≥65 | No | self-report | **AP**  never  former  current  1-19 pack-years  20-39 pack-years  ≥40 pack-years  **CP**  never  former  current  1-19 pack-years  20-39 pack-years  ≥40 pack-years | **AP**  1.00  1.25 (0.97, 1.61)  1.02 (0.81, 1.28)  0.86 (0.65, 1.15)  1.32 (1.01, 1.73)  1.25 (0.91, 1.70)  **CP**  1.00  1.59 (1.02, 2.47)  1.64 (1.11, 2.41)  1.46 (0.92, 2.30)  1.51 (0.92, 2.30)  2.03 (1.23, 3.34) |
| Sadr-Azodi, 2012, Sweden [34] | Swedish Mammography Cohort and Cohort of Swedish Men | 84,667 | 541 | 12 yrs | 46-84 | Yes | self-report | never  former  current | **AP** 1.00  1.19 (0.97, 1.46)  1.33 (1.07, 1.66) |
| Tolstrup, 2009, Denmark [35] | Copenhagen City Heart Study | 17,905 | total 235 AP 160 other 97 | 20.2 yrs | 52 | No | self-report | **Total**  never  former  1-14 g/d tobacco  15-24 g/d tobacco  ≥25 g/d tobacco  **AP**  never  former  1-14 g/d tobacco  15-24 g/d tobacco  ≥25 g/d tobacco  **Other**  never  former  1-14 g/d tobacco  15-24 g/d tobacco  ≥25 g/d tobacco | **Total**  1.0  1.7 (1.0, 2.7)  1.5 (0.9, 2.5)  2.5 (1.5, 3.9)  3.3 (1.9, 5.8)  **AP**  1.0  2.3 (1.3, 4.1)  2.0 (1.1, 3.6)  2.8 (1.5, 5.0)  3.8 (1.9, 7.5)  **Other**  1.0  0.9 (0.4, 2.0)  1.1 (0.5, 2.3)  2.0 (1.0, 4.1)  3.3 (1.5, 7.3) |
| Lindkvist, 2008, Sweden [23] | The Malmö Preventive Project | 33,346 | 179 | NA | ≥45 | No | self-report | never  former  current  <20 cigarettes/d  20-29 cigarettes/d  ≥30 cigarettes/d | **AP** 1.00  1.09 (0.66, 1.80)  2.14 (1.48, 3.09)  1.84 (1.19, 2.85)  3.19 (2.03, 5.00)  2.87 (1.57, 5.24) |
| Morton, 2004, US [36] | Kaiser Permanente Medical Care Program Cohort | 129,000 | total 439 | 12 yrs | NA | NA | self-report | never  former  current | **Total** 1.00  1.48 (1.08, 2.04)  2.08 (1.50, 2.88) |
| **Alcohol** | | | | | | | | | |
| Prizment, 2015, US [24] | Iowa Women’s Health Study | 36,436 | 511 | NA | ≥65 | No | self-report | non-drinker  drinker  0.5-3.0 g/d  >3.0 g/d | **AP** 1.00  0.85 (0.70, 1.02)  0.79 (0.62, 1.00)  0.91 (0.72, 1.15) |
| Sadr-Azodi, 2011, Sweden [39] | Swedish Mammography Cohort and Cohort of Swedish Men | 68,158 | 513 | 10 yrs | 46-84 | No | self-report | per 60g on single occasion  wine  beer  spirits | **AP**  0.86 (0.42, 1.75)  0.92 (0.47, 1.81)  1.59 (1.16, 2.19) |
| Kristiansen, 2008, Denmark [40] | Copenhagen City Heart Study | 17,905 | total 268  AP 171  other 97 | 20.1 yrs | NA | No | self-report | **Total**  0 drinks/wk  1-6 drinks/wk  7-13 drinks/wk  14-20 drinks/wk  21-34 drinks/wk  35-48 drinks/wk  >48 drinks/wk  **AP**  0 drinks/wk  1-6 drinks/wk  7-13 drinks/wk  14-20 drinks/wk  21-34 drinks/wk  35-48 drinks/wk  >48 drinks/wk  **Other**  0 drinks/wk  1-6 drinks/wk  7-13 drinks/wk  14-20 drinks/wk  21-34 drinks/wk  35-48 drinks/wk  >48 drinks/wk | **Total**  1.0  1.1 (0.8, 1.6)  1.2 (0.8, 1.8)  1.3 (0.8, 2.1)  1.3 (0.7, 2.2)  2.6 (1.4, 4.8)  3.0 (1.6, 5.7)  **AP**  1.0  1.2 (0.7, 1.8)  1.4 (0.9, 2.3)  1.3 (0.7, 2.4)  1.7 (0.9, 3.2)  3.5 (1.8, 7.1)  3.3 (1.5, 7.3)  **Other**  1.0  1.2 (0.7, 2.3)  1.2 (0.6, 2.4)  1.5 (0.7, 3.2)  1.3 (0.6, 3.1)  2.7 (1.1, 6.6)  3.3 (1.3, 8.3) |
| **Gallstone disease** | | | | | | | | | |
| Lin, 2014, Taiwan [43] | the National Health Interview Survey | 35,642 participants | total 66 | 3 yrs | mean: 43.7 | NA | medical record | gallstone | **AP** 17.04 (9.82, 29.58) |
| Urushihara, 2012, Japan* [27] | hospital administrative database in Japan | 1375 cases, 5469 controls | 1375 | NA | mean: controls 62 DM 67 | No | medical record | gallstone | **AP** 14.29 (11.60, 17.62) |
| Girman, 2010, UK* [30] | General Practice Research Database | 2,984,755, 5.0% with T2DM | 2735 | NA | mean 47.3 | No | medical record | gallbladder disease | **AP** 1.56 (1.26, 1.93) |
| Garg, 2010, US* [29] | a large medical and pharmacy claims database | 38,615 diabetes patients, 748,041 controls | 508 | NA | mean 52 | No | medical record | gallstone | **AP** 6.2 (4.5, 8.6) |

* Record linkage studies.

Abbreviations: AP, acute pancreatitis (ICD-10 K85); CP, chronic pancreatitis (ICD-10 K86.1); other, other diseases of the pancreas (ICD-10 K86); RR, relative risk.
