# Supplementary figures and images for: Metabolic and lifestyle risk factors for acute pancreatitis in Chinese adults: A prospective cohort study of 0.5 million people
Source: PLoS Med. 2018 Aug 1;15(8):e1002618. doi: 10.1371/journal.pmed.1002618 (PMC6070164; doi:10.1371/journal.pmed.1002618)

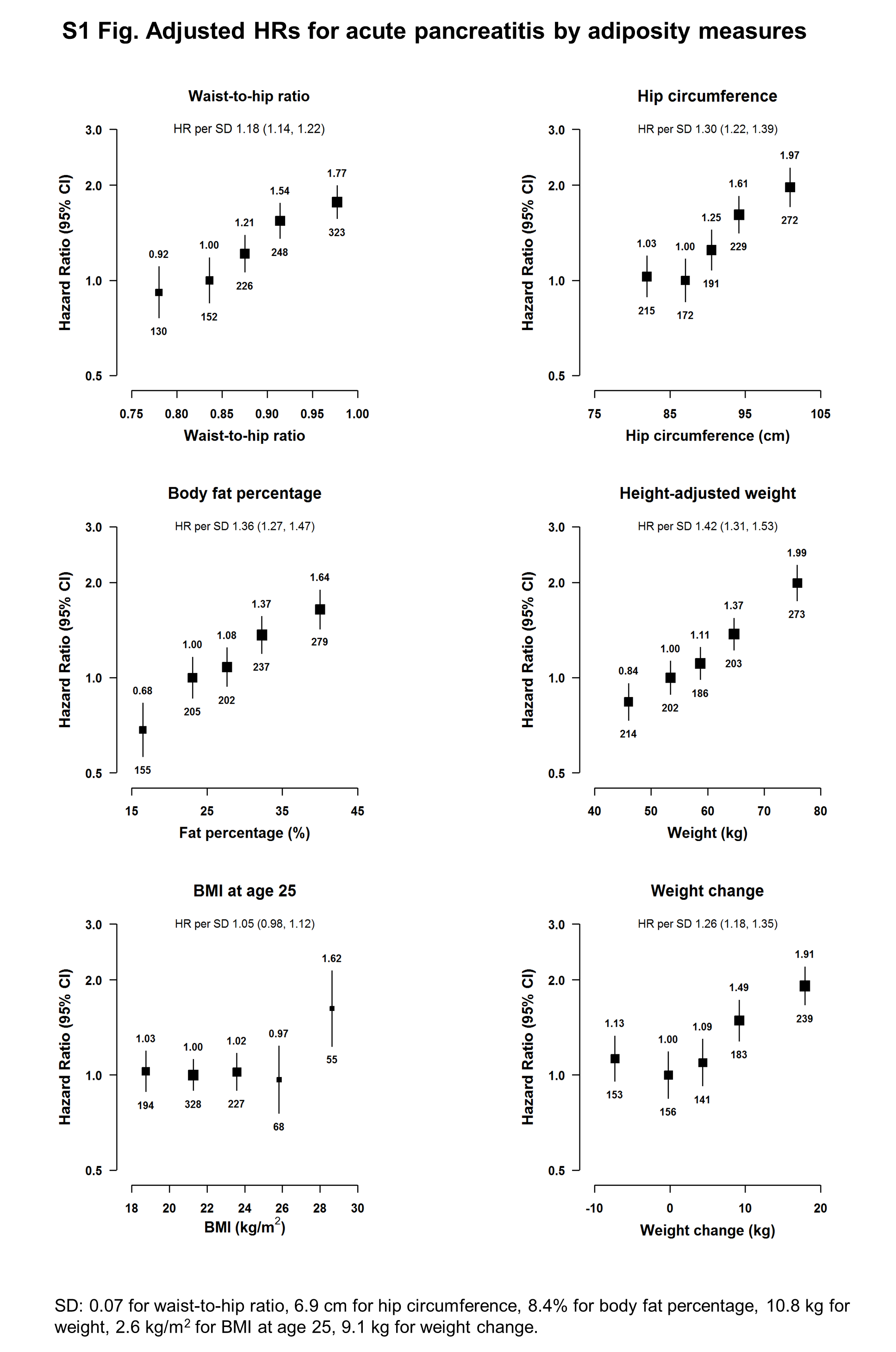

Supplement: S1 Fig — HR, hazard ratio. (TIF) [file pmed.1002618.s011.tif]

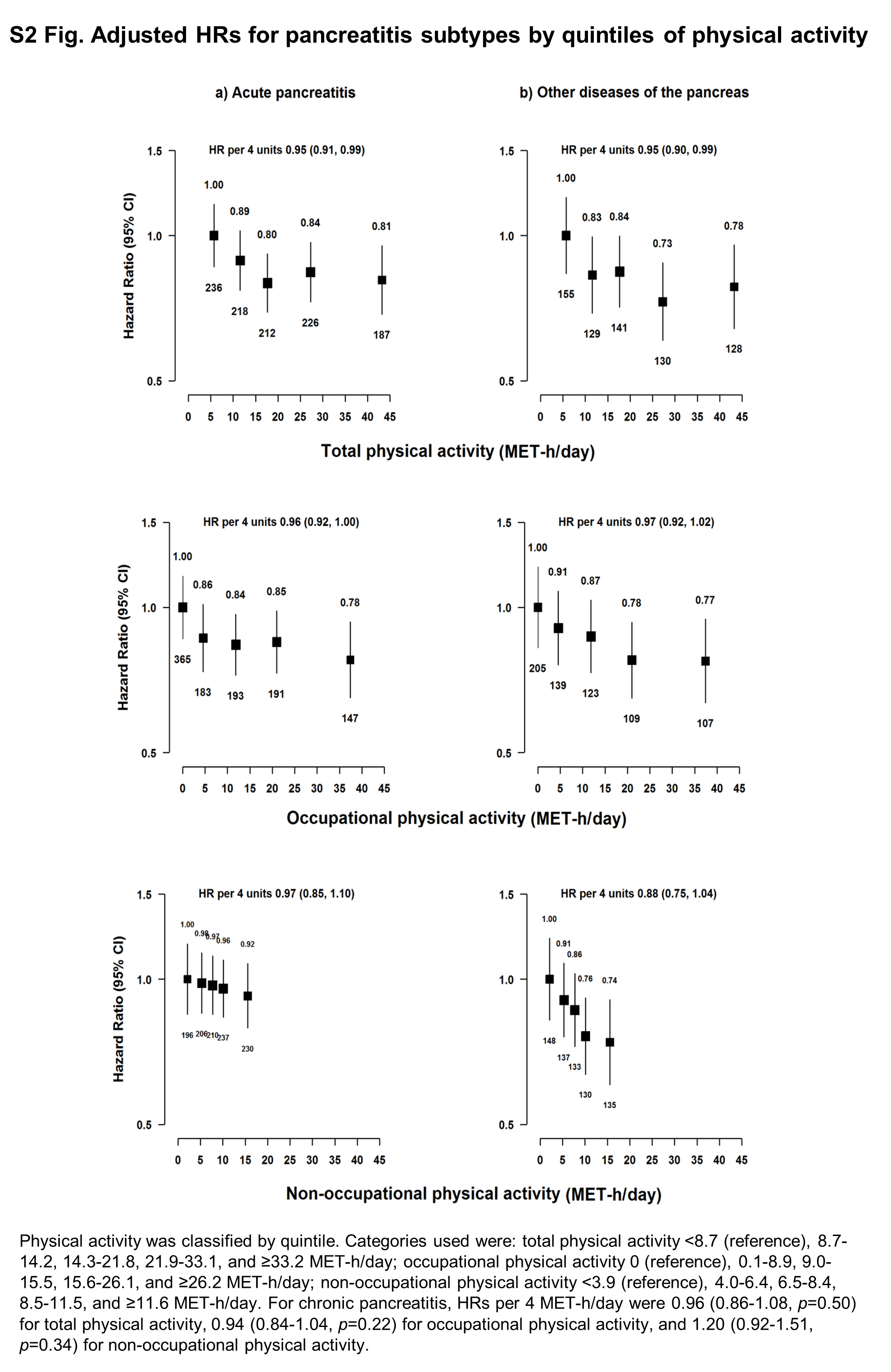

Supplement: S2 Fig — HR, hazard ratio. (TIF) [file pmed.1002618.s012.tif]

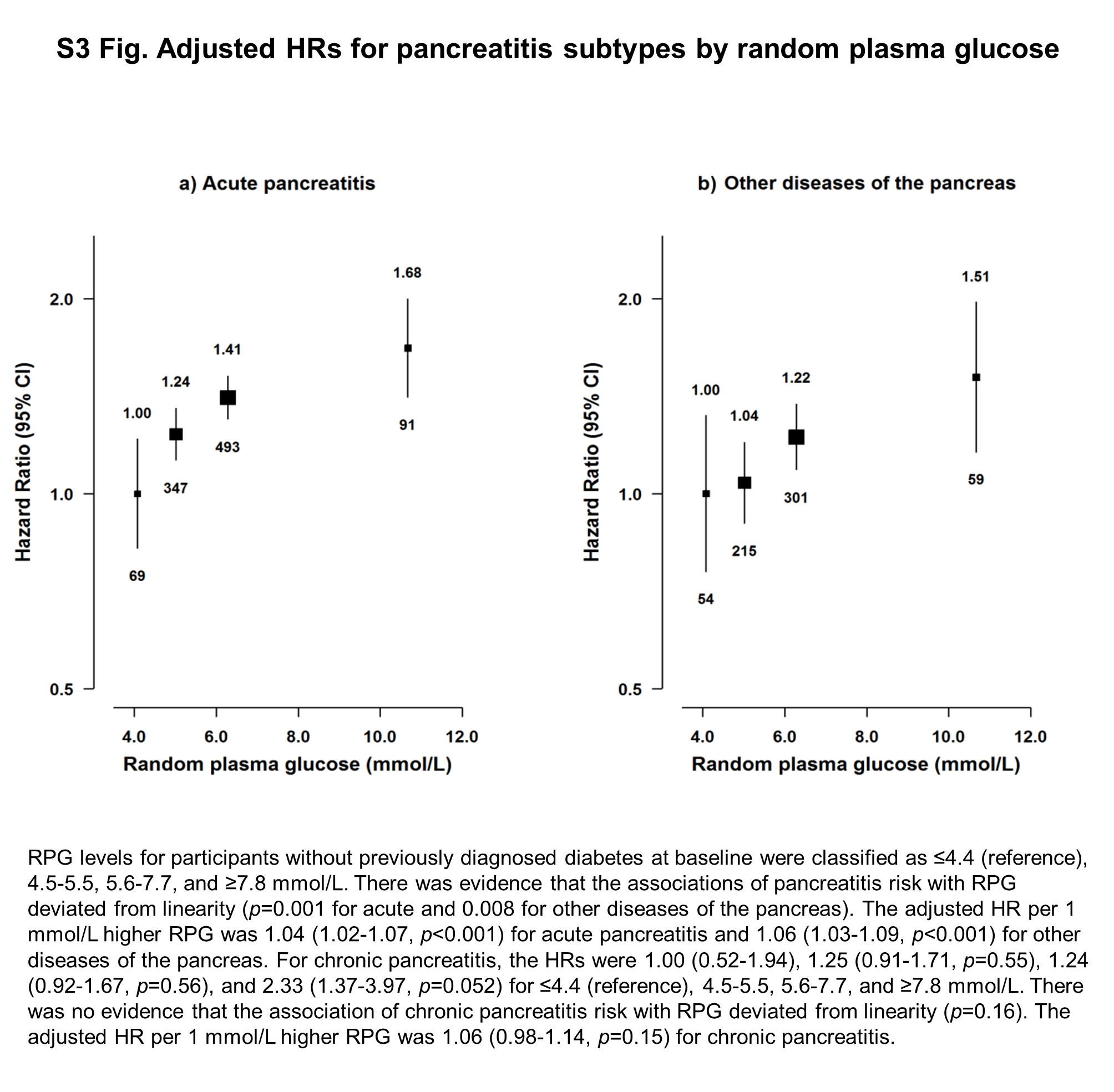

Supplement: S3 Fig — HR, hazard ratio; RPG, random plasma glucose. (TIF) [file pmed.1002618.s013.tif]
